# Pediatric subspecialty pipeline: aligning care needs with a changing pediatric health care delivery environment

**DOI:** 10.1038/s41390-023-02599-x

**Published:** 2023-05-04

**Authors:** Mona Patel, Jean L. Raphael

**Affiliations:** 1 Department of Pediatrics, Children’s Hospital Los Angeles, Los Angeles, CA USA; 2 Center for Child Health Policy and Advocacy, Baylor College of Medicine, Houston, TX USA

In 2013, the American Academy of Pediatrics Committee on Pediatric Workforce released its policy statement describing critical workforce shortages in pediatric medical and surgical subspecialties that impact the provision of quality health care for US children.^1^ The pediatric subspecialty workforce increasingly encounters the pressures of pediatric access to care, changing care delivery to more complex care at academic institutions, and a shift toward inclusion of social risk factors in care delivery. Literature has advanced reviews of pediatric specialty fellowship positions, including their increased albeit disparate availability over time, comparisons of fill rates, and underlying reasons for specialty fellowship selection by applicants.^2^ Ultimately, access to pediatric subspecialty workforce is further constrained by the fact that majority of pediatric specialty practice exists at academic centers leaving widely disparate geographic shortage areas, especially in rural and underserved regions.^3^

Academic institutions deliver a majority of pediatric subspecialty care compared with community sites,^4^ and some of these organizations also care for larger proportions of publicly insured populations. Poor reimbursement in the Medicaid system for pediatric health care further compounds specialty access in creating challenges in pediatric specialty reimbursement. Compensation that relies on work relative value unit function impacting those with high clinical workloads also adds to burnout and dissatisfaction amongst pediatric specialists working in health care today. Specialty pediatric access must also include a review of the demands upon this service from referring primary care pediatricians and caregivers, ensuring the appropriateness of referrals to subspecialists and timely return of continued general patient care to primary care. Many pediatric specialists, often caring for our most complex pediatric patients, often deliver primary care including immunizations and serve as the first-line care sites for common pediatric complaints such as fever and cough. While the expansion of medical home concepts may be beneficial to the patient and family, these additional services are often not reimbursed. Catenaccio et al. reviewed a comparison of the lifetime earning potential of pediatric specialists compared with general pediatrics, revealing a high medical education loan burden, lower overall earning potential, and years of lack of meaningful income during fellowship leading to negative financial impacts in most pediatric specialties, especially non-procedural based specialties.^5^

Finally, academic institutions hold value in excellence in clinical care, education, advocacy, and research; the latter three elements are not directly tied to clinical revenue streams, hence usually unsupported, adding to the circuitous impacts of health care finance ultimately on pediatric specialty access. The unique contributions of physician-scientists who have completed pediatric subspecialty fellowships are further compromised, with pediatric specialties report <10% of pediatric specialists devoting >50% of time in research.^6^ Given these multifactorial complexities in having a sufficient pediatric workforce enabling the provision of quality health care for US Children, in 2020, the Association of Medical School Pediatric Department Chairs (AMSPDC) launched a collaborative, multi-organizational initiative called Pediatrics 2025: The AMSPDC Workforce Initiative, focused on strengthening the pediatric workforce.^7^ This taskforce, comprised of leading pediatric organizations with prominent roles in pediatric clinical care delivery; policy; education, advocacy, and research, continues to work in four domains, including educational paradigms in attracting diverse trainees into undersubscribed pediatric subspecialties, workforce data needs and access, economic strategy, and attracting high-quality medical students into pediatrics.

In this article by Freed et al., the authors compared National Resident Matching Program (NRMP) match rates with fill rates reviewed by the American Board of Pediatrics (ABP). The authors reviewed 14 pediatric subspecialties from 2008 to 2020 and compared the number of open positions, the NRMP match rate, the ABP fill rate, and the actual number of matriculating pediatric subspecialty fellows for those years. They found that the ABP data source should be prioritized when assessing pediatric subspecialty pipeline, as the former data depict an incomplete lens on actual fill rates for pediatric specialties. In the review, there was a comparison of pediatric medical specialties, and in using the ABP fill rate, and there has been an absolute increase in the number of pediatric medical subspecialty fellowship positions since 2008, except for adolescent medicine. The authors describe that since the NRMP match results earlier in the academic year, typically in November or December with specialty match, there remains a gap in time where unmatched positions can be “scrambled” to fill later in the academic year. In comparison, the ABP fill rate is finalized closer to matriculation dates, typically June of each academic year, and is therefore more indicative of the health of the specialty pipeline.

The authors describe the importance of recognizing the impacts of data source on decision making, since ultimate funding and policy targets are made with data-driven information. The authors further describe that there continues to be a disparity in the increased positions across the pediatric medical specialties, with procedural specialties such as emergency medicine and critical care having larger increases in a number of positions compared to non-procedural pediatric subspecialties such as Endocrinology, Developmental & Behavioral Pediatrics and Infectious Diseases. It was notable that in this article, the authors reviewed pediatric medical specialties only and did not address pediatric surgical specialty pipeline. In another recent review, there is a question about the pediatric surgical pipeline, with a relative decrease in match rates due to less operation exposure and a need to review training curricula. The authors acknowledge that there has not been consensus on how many pediatric specialties are needed across the US, which impacts ultimate policy and funding targets for pediatric specialty pipeline expansion. There is also discussion that some pediatric specialty positions may go unfilled due to a recent increase in program size, or institutional needs, or funding for a newer program.

Federal and state policy efforts have prioritized the use of physician loan repayment programs to increase interest in pediatric specialty programs in hopes of decreasing the financial impact of selecting pediatric specialty focus. The recent 2023 omnibus governmental spending package included $10 million for the Pediatric Specialty Loan Repayment Program, which was double the amount compared to 2022. With the continued disparity in fill rates for non-procedural, pediatric medical specialties, critically thinking about selection criteria for the first round of the Health Resources & Services Administration awards should be considered when addressing disparities regionally. Similarly, the National Institutes of Health have implemented a Pediatric Research Loan Repayment Program, understanding the negative impacts that financial strains of prolonged research training programs bring upon ultimate selection.

Broadening the goal toward enhancing pediatric specialty access must be considered rather than simply taking into account pediatric specialty workforce numbers. As an example, in order to truly understand pediatric specialty access, it will require balancing an appreciation of pediatric operational supply and demand. We have discussed the insufficient supply of pediatric specialty workforce, but principles of demand must be considered, including the availability of high-quality primary care with integrated team-based models integrating social and environmental risk assessments into clinical care, and understanding the true need of high-impact specialty care. With continued challenges in discharging patients from specialty care back to primary care and review of follow-up specialty visit timelines, upwards of 30–50% of specialty care visits are deemed inappropriate (either parent-induced demands or inappropriate discharge).^8^ Demands upon specialty access were especially increased during the COVID-19 pandemic, which federal and state care delivery changes allowed for innovation such as telehealth, potential e-consult methods of physician-to-physician asynchronous curbside supports, and collaborative primary care and pediatric specialty care models should continue in the post-pandemic, changing health care delivery era.^9^ By ending public health emergency periods at the federal and state level, certain leniencies and reimbursement for such enhanced access, such as telehealth provisions, should continue.

In consideration of reimbursement opportunities for pediatric specialty care, advocacy to review current procedural terminology codes drawn down from Centers for Medicaid & Medicare Services and allow for parity in reimbursement for non-procedural and procedural codes can aid with financial strain at the institutional level.^10^ In addition, a thoughtful review and support for enhanced Medicaid reimbursement will allow academic centers where a majority of pediatric specialty care is delivered with disproportionately higher Medicaid populations served to enable greater support for academic specialists. Finally, moving from Fee for Services reimbursement models toward value-based payment structures allows for the recognition of the physician and integrated team members such as social work and community health workers add cost-effective models in care delivery, especially for impacted sites delivering care for a high proportion of publicly insured patients and families. Continued support for the National Academy of Sciences, Engineering and Medicine review of pediatric specialty workforce and its impact on child health and well-being will hopefully enable further insight into meaningful investments required to allow for thoughtful support and expansion to appropriate access to our pediatric specialty workforce.
